# Transarterial Chemoembolization for Hepatocellular Carcinoma: Why, When, How?

**DOI:** 10.3390/jpm12030436

**Published:** 2022-03-10

**Authors:** Evgenia Kotsifa, Chrysovalantis Vergadis, Michael Vailas, Nikolaos Machairas, Stylianos Kykalos, Christos Damaskos, Nikolaos Garmpis, Georgios D. Lianos, Dimitrios Schizas

**Affiliations:** 1 Second Propaedeutic Department of Surgery, National and Kapodistrian University of Athens, General Hospital of Athens “Laiko”, AgiouThoma 17, 11527 Athens, Greece; nmachair@gmail.com (N.M.); kykalos@gmail.com (S.K.); x_damaskos@yahoo.gr (C.D.); nikosg22@hotmail.com (N.G.); 2 Department of Radiology, General Hospital of Athens “Laiko”, AgiouThoma 17, 11527 Athens, Greece; chvergadis@gmail.com; 3 First Department of Surgery, National and Kapodistrian University of Athens, General Hospital of Athens “Laiko”, AgiouThoma 17, 11527 Athens, Greece; mike_vailas@yahoo.com (M.V.); schizasad@gmail.com (D.S.); 4 Department of Surgery, University Hospital of Ioannina, 45110 Ioannina, Greece; georgiolianos@yahoo.gr

**Keywords:** hepatocellular carcinoma, transarterial chemoembolization, cirrhosis, liverneoplasm

## Abstract

Hepatocellular carcinoma (HCC) is the most common primary liver malignancy. It is principally associated with liver cirrhosis and chronic liver disease. The major risk factors for the development of HCC include viral infections (HBV, HCV), alcoholic liver disease (ALD,) and non-alcoholic fatty liver disease (NAFLD). The optimal treatment choice is dictated by multiple variables such as tumor burden, liver function, and patient’s health status. Surgical resection, transplantation, ablation, transarterial chemoembolization (TACE), and systemic therapy are potentially useful treatment strategies. TACE is considered the first-line treatment for patients with intermediate stage HCC. The purpose of this review was to assess the indications, the optimal treatment schedule, the technical factors associated with TACE, and the overall application of TACE as a personalized treatment for HCC.

## 1. Introduction

Hepatocellular carcinoma (HCC) is the most common primary liver malignancy, representing approximately 90% of primary liver cancers [1]. HCC constitutes a major health problem with an increasing incidence over the years in both developed and developing countries [1]. HCC is principally associated with liver cirrhosis and chronic liver disease. Approximately, one third of cirrhotic patients will develop HCC eventually in their lifetime, with a 1-year rate of 1–8% [2]. The major risk factors for the development of HCC are viral infections (hepatitis B and C virus-HBV, HCV), alcoholic liver disease (ALD), and non-alcoholic fatty liver disease (NAFLD). Viral hepatitis represents the most common risk factor for HCC. Nevertheless, vaccination for HBV and antiviral therapy for HBV/HCV have reduced the incidence of HCC in countries with an organized vaccination program, while NAFLD-related cirrhosis continues to increase, representing the leading cause of HCC in the developed world [3].

HCC is usually diagnosed during routine examination, since most cirrhotic patients in developed countries enter a screening program. Unfortunately, many countries do not have an organized screening program and HCC patients are often diagnosed in advanced stage. All high-risk patients for HCC should be monitored with ultrasonography (US) and alpha-fetoprotein (AFP) measurement every 6 months [1]. Nevertheless, since US is considered operator-dependent and AFP is often normal in early stage, computed tomography (CT) and magnetic resonance imaging (MRI) are used to characterize nodules bigger than 10 mm.

Over the years, several HCC classification systems have been developed. Most of them include parameters such as tumor stage, liver function impairment, patient’s performance status, and recommended treatment strategy. The Barcelona Clinic Liver Classification (BCLC) is a staging system widely accepted worldwide [4]. BCLC stratifies patients according to the natural history of the disease, selecting the best candidates for the best therapies [1,5].

The treatment choice depends on multiple variables such as tumor burden, liver function, and patient’s performance status. Surgical resection, transplantation, ablation, transarterial chemoembolization (TACE), and systemic therapy are potentially useful treatment strategies. All HCC patients should be referred to a multidisciplinary team for treatment option evaluation [6]. Liver resection should be considered in the setting of preserved liver function. Patients with early lesions (single < 2 cm) in the absence of portal hypertension can benefit from surgical resection [7]. HCC patients in early stage (BCLC stage 0 and A) with underlying cirrhosis and portal hypertension can be treated with liver transplantation (LT) [8]. Locoregional ablation treatments such as radio-frequency ablation (RFA) and microwave ablation (MWA) are mainly available options for patients who are not fit for surgery but also as a bridge to LT [6]. Arterially directed therapies (TACE and transarterial radioembolization-TARE) are the first-line treatment in patients with intermediate stage (large multi-nodular tumors, relatively preserved liver function, cancer confined to the liver) [9]. Stereotactic body radiation therapy (SBRT) is another treatment option for patients with non-resectable HCC [10]. SBRT causes tumor necrosis by delivering high doses of radiation to target lesions with great accuracy [11]. Finally, systemic therapy with the use of sorafenib (an oral multikinase inhibitor) for patients in advanced stage, with portal invasion and extrahepatic spread, can improve overall survival [12]. Patients in terminal stage can be offered the best supportive care. HCC is associated with significant financial burden, affecting both patients and health care systems, especially in low-income countries where medical resources are limited. Therefore, cost-effectiveness studies are expected in priority to determine the optimal treatment strategy for HCC [13].

## 2. Treatment Procedure

TACE treatment involves the infusion of highly concentrated dose of chemotherapy through selective catheterization of the arterial branch feeding the tumor. The embolization of the tumor microcirculation following the infusion results in prolonged cytotoxic effect, minimizing the systemic toxicity of chemotherapy [14]. The dual blood supply to the liver, from the hepatic artery and the portal vein, makes TACE, as well as arterially directed therapies in general, possible, and protects healthy liver tissue from ischemia. on the contrary to the normal parenchyma that derives blood supply mostly from the portal vein, tumor cells get blood flow mainly from the hepatic artery [15].

### 2.1. cTACE vs. DEB-TACE

There are two types of TACE techniques: conventional TACE (cTACE) and TACE with drug-eluting beads (DEB-TACE). cTACE uses a cytotoxic agent such as doxorubicin, epirubicin, mitomycin, or cisplatin, followed by the infusion of Lipiodol, an oily radio-opaque agent, as a chemotherapeutic carrier, as well as an embolic material [16]. Other embolic agents commonly used are degradable starch microspheres (DSM), collagen, and gelatine sponge (Gel-foam). DEB-TACE uses non-resorbable embolic microspheres loaded with chemotherapy drugs that are capable of releasing the agent in a sustained manner [17].

It is still controversial whether the one technique is superior to the other. In a meta-analysis performed by Zou et al., DEB-TACE appeared to have an improved complete response rate and overall survival rate when compared to cTACE. Furthermore, DEB-TACE patients reported decreased common adverse events than cTACE, with no statistically important difference between the two therapies on serious adverse events [18]. Chen et al. and Han et al. reached a similar conclusion; the overall survival rates were significantly higher in the DEB-TACE group, with no statistically significant difference in tumor response and treatment-related adverse events [19,20]. On the contrary, two meta-analyses concluded that cTACE and DEB-TACE had similar therapeutic results, overall survival, and adverse events rates, underlying the need for further research with high-quality studies [21,22].

**Figure 1:**
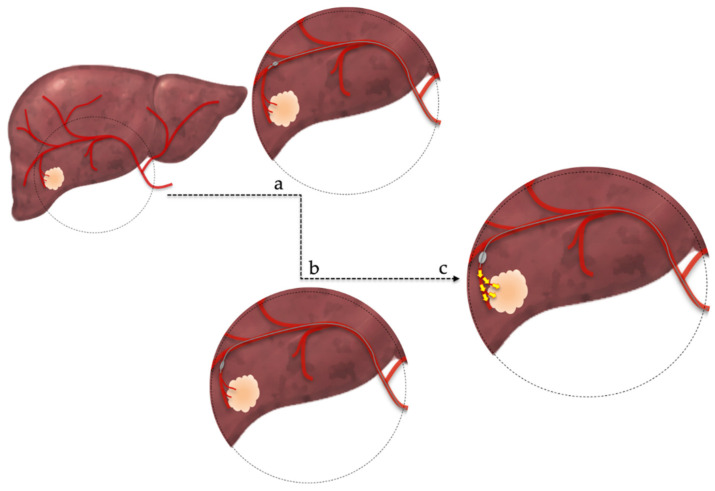
Balloon-occluded transarterial chemoembolization technique. (**a**,**b**): superselective catheterization of the arterial branch feeding the tumor, (**c**): occlusion of feeding artery by infletion of a microballoon catheter and subsequent administration of chemotherapeutic regimen.

Recently, a new method of arterial occlusion during TACE has been proposed [23]. This new method, named balloon-occluded transarterial chemoembolization (B-TACE), uses a balloon micro-catheter in order to selectively occlude the arterial micro-circulation of the tumor (Figure 1) [24,25]. The advantages of this method are the prevention of embolic agents’ leakage and the increased accumulation of Lipiodol emulsion within the tumor that may enhance treatment success due to prolonged cytotoxic effect [23]. The current literature suggests a potential advantage of B-TACE, compared to DEB-TACE, for patients with large tumors, but further studies must be conducted to reach safe conclusions [26].

### 2.2. Patient Selection

In accordance with the BCLC guidelines, TACE is currently considered the first-line treatment for selected patients with HCC in the intermediate stage (BCLC stage B). This stage includes patients with unresectable, multinodular tumors without vascular invasion or extrahepatic spread.Moreover, these patients present heterogeneous features, in terms of tumor burden and liver function (Child-Pugh A or B) [27]. Therefore, not all intermediate-stage HCC patients benefit the same from TACE, since this heterogeneity makes the behavior of the tumor difficult to predict. Various systems for subclassification of intermediate-stage HCC have been proposed. Among these systems, up-to-seven criteria, originally used to predict the prognosis of HCC patients undergoing LT, were also proposed to subclassify patients within BCLC-B stage [28]. These criteria include the sum of the diameter of the largest tumor (in cm) and the number of tumors [29].In an attempt to sub-classify this heterogeneous group of patients, the current data support that patients of intermediate stage that may benefit most from TACE are those with Child-Pugh scores ≤7, preserved performance status (PS 0), large multinodular tumor burden (within the up-to-seven criteria) but not bulky, and being without vascular invasion or extra-hepatic disease [30].

TACE can also be beneficial for some patients beyond BCLC stage B. In patients with early stage disease (BCLC A) who are unsuitable for surgery or locoregional ablation, TACE consists a safe and effective option with a high response rate and very good outcomes [31]. TACE can also be performed prior to liver transplantation as a bridging treatment while the patient is on the waiting list or as downstaging treatment to within Milan criteria [16]. Finally, some patients with advanced disease can be treated with TACE. In those patients, TACE is possible if they have segmental or sub-segmental portal vein thrombosis and the treatment is selective. A recent meta-analysis showed improved overall survival in the TACE group and better tumor response when compared with conservative treatment [32].

TACE unsuitability is defined as each one of the following three clinical conditions: (adapted from Asia-Pacific Primary Liver Cancer Expert Consensus [33])

▪ Unlikely to respond to TACE (confluent multinodular type, massive type, poorly differentiated HCC, extranodular growth and others)
▪ Likely to develop TACE failure/refractoriness (beyond up-to-seven criteria)
▪ Likely to cause deterioration of liver function (beyond up-to-seven-criteria, albumin/bilirubin-ALBI grade 2).

### 2.3. Contraindications and Adverse Effects

There are limited contraindications to TACE therapy, mainly concerning the residual liver function or impaired portal blood flow. Advanced cirrhosis (Child-Pugh C), liver failure, total bilirubin > 3 mg/dL, presence of extrahepatic disease, complete portal vein thrombosis, uncorrectable coagulopathy, and the presence of high-flow arterioportal or arteriovenous shunts represent some of them [27]. Severe atherosclerotic disease, renal insufficiency, and allergy to contrast material are considered relative contraindications [34].

Even though TACE is considered a relatively safe procedure, several adverse events have been documented. Since the hepatic artery also supplies the biliary plexus, TACE can cause ischemic complications such us pancreatits, cholecystitis, andbile duct necrosis, but also liver and biliary injuries, liver abscess formation, and less selective embolization resulting in liver failure [15].The TACE-related mortality rate is considered low (<1%) [35]. Most commonly, up to 47% of patients treated with TACE develop a clinical syndrome mediated by an inflammatory response, as a result to cytokines’ release. This post-embolization syndrome (PES) presents with fever, right upper quadrant abdominal pain, and nausea with or without vomiting [36]. PES is associated with prolonged hospital stays and recurrent admissions, but it is also considered an early predictor of worse overall survival [36,37]. Prophylactic administration of steroids and 5-HT3 receptor antagonists has been used to prevent PES [38]. In a retrospective study by Haohao et al., the lipiodol + dexamethasone emulsion significantly reduced the incidence rate of post-embolization syndrome [39].

## 3. Prognostic Scores

Over the last decade, several prognostic scores have been developed to predict the results of TACE treatment. (Table 1) The criteria for TACE refractoriness has been established for the first time in the world by the Japan Society of Hepatology (JSH) in 2011 [40]. TACE failure/refractoriness is defined by the International Expert Panel of Interventions in Hepatocellular Carcinoma (EPOIHCC) as no response after three or more TACE procedures within a period of six months [41]. The incidence of TACE failure/refractoriness is considered to be quite high, ranging from 37 to 49.3% [42]. Therefore, it is essential to identify prognostic factors in order to differentiate patients who would benefit or not from repeated TACE procedures.

The Hepatoma arterial-embolization prognostic (HAP) score stratifies patients into four groups. Patients are divided according to their albumin, bilirubin, AFP levels, and the size of dominant tumor. One point is assigned if albumin < 36 g/dL, bilirubin > 17 μmol/L, AFP > 400 ng/mL, or size of dominant tumor > 7 cm. The HAP score is calculated by the sum of these points, and patients are classified into low-(HAP A, score 0), intermediate-(HAP B, score 1), high-(HAP C, score 2), or very high-(HAP D, score > 2) risk groups. The median survival for the groups A, B, C, and D was 27.6, 18.5, 9.0, and 3.6 months, respectively [43].

The ART score (Assessment for Retreatment with TACE) was created to differentiate patients who would benefit from a second TACE procedure. The creators of the score conducted a study by dividing patients in two groups based on radiologic tumor response after the first TACE, whether there was an increase of serum AST (aspartate aminotransferase) >25%, and whether there was an increase of Child-Pugh score of 1 or ≥2 points. The two groups (group one: ART score between 0–1.5 points, group two: ART score ≥ 2.5 points) showed significantly different median overall survivals (23.7 versus 6.6 months). Therefore, the study concluded that a higher ART score was associated with worse prognosis and major adverse events and that those patients may not benefit from further TACE [44].

The STATE-score (Selection for TrAnsarterial chemoembolization TrEatment) was created to identify patients who are unsuitable for first-time TACE. Hucke et al. divided patients in two groups (<18, ≥18 points) according to their albumin and CRP levels and whether they are in or beyond the up-to seven-criteria (if the sum of the diameter of the largest tumor and the number of tumors is less than seven). The median survival was 5.3 months for the first group (<18 points) and 19.5 months for the second (≥18 points). The researchers concluded that a lower STATE-score was associated with increased mortality after TACE-1 [45]. They also combined the STATE and ART score, namely START strategy to identify the best candidates for multiple TACE [45].

The ABCR score (Alpha-fetoprotein, BCLC, Child-Pugh, and Response), similarly to the ART score, was developed to identify appropriate patients who would benefit from TACE retreatment. This score, ranging from minus three to six, includes four parameters: AFP, BCLC, Child-Pugh increase by more than two points, and radiological response. The analysis conducted by Abhoute et al. in order to validate the score revealed that patients with ABCR score ≥4 after the first TACE procedure had a median overall survival < 5.1 months and probably would not benefit from repeated TACE [46].

The CHIP score (Chiba HCC in Intermediate-stage Prognostic), helps stratify patients within the heterogeneous stage B. This score, with a range between zero and seven, is defined by the sum of three subscale scores: Child-Pugh, number of lesions, and HCV-RNA positivity. According to their sum, patients were stratified in five groups (zero to two points, three points, four points, five points, and six to seven points). The creators of the score came to the conclusion that each group corresponds to different prognosis (65.2, 29.2, 24.3, 13.1, and 8.4 months median OS, respectively) [47].

The Munich-TACE score (M-TACE) uses the values of bilirubin, international normalized ratio, C-reactive protein, creatinine, and AFP, as well as tumor extension (size and number of nodules, vascular invasion, metastasis) to divide patients in three subgroups. M-TACE was validated in a cohort analysis revealing that patients in group one (zero to nine points) had a median survival of 35.2 months, patients in group two (10–13 points) had a median survival of 16.9 months and finally patients in group 3 (>13 points) had a median survival of 8.6 months [48].

Recently, a novel prognostic score has been developed. This new stratification model, named ‘six and twelve’ score, divides patients in three groups, according to the sum of tumor size (diameter of the largest nodule) and tumor number (group one: sum ≤ 6, group two: 6 < sum ≤ 12, group three: sum > 12). The creators of the score conducted a validation analysis that resulted in distinct prognosis. The median survival rates for each group were 49.1 months, 32 months, and 15.8 months, respectively [49]. In contrast to previous scores, this study included only “ideal” TACE candidates, defined as treatment-naïve patients, unresectable BLCL stage A or stage B, with Child-Pugh scores between A5 and B7 and performance status 0.

In 2020, Han et al. created two new prognostic scores: the pre-TACE model (“Pre-TACE-Predict”) and the post-TACE model (“Post-TACE-Predict”). The parameters included in point assigning were tumor number and size, alpha-fetoprotein, albumin, bilirubin, vascular invasion, cause, and response, as assessed by mRECIST criteria. According to their score, patients were classified in four distinct risk categories, with median overall survivals ranging between seven months to more than four years [50].

## 4. Combined Treatments

Locoregional ablation therapies, such as radiofrequency ablation (RFA) and microwave ablation (MWA), are considered suitable alternatives to early stage HCC patients (BCLC 0 and A) who are not fit for surgery (resection or transplantation) [51]. Using a needle electrode, RFA creates an electrical current in the radiofrequency range in order to provoke heat-based thermal cytotoxicity. By achieving a temperature range between 60–100 °C, this electrical current can cause instant thermocoagulation necrosis [52]. RFA is most suitable for lesions up to 3 cm, with a margin of 0.5–1 cm of liver parenchyma needed in order to include any possible microscopic extension of the tumor [53]. During ablation, energy disperses from the target lesion because of the cooling effect of hepatic blood flow. Due to this phenomenon, known as the heat-sink effect, RFA is less effective when the tumor is located near large vessels [54]. MWA causes tissue necrosis by using high frequency electromagnetic energy. This energy leads a continuous rotation of dipole molecules, primarily water, in the microwave’s oscillating electric field, causing coagulation necrosis [53]. MWA, when compared to RFA, can achieve higher temperature at the target lesion more rapidly and its efficacy is less influenced by heat-sink effect. This results in expansion of the ablation zone, allowing MWA to treat lesions up to 8 cm [53,55].

### 4.1. TACE & RFA

In a study comparing the safety and efficacy of either TACE + RFA, TACE, or RFA monotherapy in patients with small HCC (≤3 cm), tumor response of TACE-RFA appeared to be similar to that of RFA and better than TACE monotherapy. Furthermore, patients receiving combination therapy reported complications and discomfort more frequently than those receiving either TACE or RFA monotherapy. Overall, the researchers concluded that regarding small HCC, patients that may benefit from TACE-RFA are those who are ineligible for RFA monotherapy due to tumor location [56]. A different study, investigating the prognostic value of RFA combined with TACE in patients with medium to large HCC (3–10 cm), concluded that combination therapy of TACE with RFA is a safe and effective treatment for those patients by delaying tumor progression and improving progression-free survival and overall survival [57]. This conclusion can be interpreted by the fact that even if the current data state that RFA is not recommended for tumors > 3 cm, TACE prior to RFA can facilitate ablation by causing hypoxia and reducing vascularity. In a randomized controlled trial conducted by Peng et al., the researchers compared the overall survival and the recurrence-free survival of HCC patients that received either TACE and RFA or RFA alone. They concluded that patients in the TACE-RFA group had better overall survival and recurrence-free survival than patients in the RFA group (hazard ratio, 0.525; 95% CI, 0.335 to 0.822; *p* = 0.002; hazard ratio, 0.575; 95% CI, 0.374 to 0.897; *p* = 0.009, respectively) [58]. The same conclusion, regarding the superiority of combined TACE/RFA treatment when compared to RFA monotherapy, was reached by a different RCT conducted in patients with recurrent HCC [59].

### 4.2. TACE & MWA

A randomized control trial by Zaitoun et al., comparing TACE or MWA monotherapy to combined treatment, concluded that combined therapy was safe, well-tolerated and more effective than TACE or MWA alone for treatment of HCC 3–5 cm. Those patients displayed lower recurrence rate after 12 months and significantly higher overall survival rate and mean progression-free survival [60]. A recent meta-analysis investigating the effectiveness of TACE + MWA versus TACE alone for unresectable BCLC stage A or B HCC < 5 cm showed that complete response, partial response, and objective response rates were significantly higher in TACE + MWA than those in TACE alone [61].

### 4.3. TACE & RFA vs. TACE & MWA

In a recent analysis, Yuan et al., comparing the efficacy and safety of TACE in combination with RFA and MWA in the treatment of middle and large primary hepatic carcinoma (≥3 cm), concluded that even if the difference in short-term efficacy, survival rates, and adverse reaction rates between the two combined methods had no statistical significance, when the tumor size was over 5 cm, the efficacy in TACE + MWA group was better than that in TACE + RFA group. Furthermore, postoperative liver function damage in the TACE + RFA group was lighter than that in TACE + MWA group [62]. A different analysis, studying the survival benefit of ablation techniques combined with TACE, reached the conclusion that concerning tumors sized 3–5 cm, TACE-MWA group showed a higher tendency to provide complete response rates than TACE-RFA, with no difference in survival rates and recurrence free survival at 1 year [63].

### 4.4. TACE & Irreversible Electroporation

Recently, a new ablative technique, called irreversible electroporation (IRE), has been introduced in liver malignancies. IRE is a non-thermal ablative treatment leading to cell death by generating pulses of high voltage and intensity of short duration and creating pores in lipid bilayer of cellular membranes [64]. With this technique, the ablation is safer near vital structures due to the non-thermal energy and the probe-defined ablative zone [65]. So far, to our knowledge, only one experimental trial has been conducted studying the efficacy of combined IRE-TACE therapy. In 2019, Isfort et al. compared the local effect of IRE followed by DEB-TACE versus IRE alone in a porcine model. The result of the trial suggested that local efficacy of IRE can be enhanced by post-IRE DEB-TACE [66].

### 4.5. TACE & Sorafenib

Sorafenib is an oral multikinase inhibitor suppressing tumor cell angiogenesis and proliferation by targeting several tyrosine kinases such as VEGF, RAF, and the PDGF receptor [67]. Sorafenib has been considered the standard of care treatment for patients with advanced stage HCC for almost a decade. Nevertheless, several newer agents have been established over the years, replacing sorafenib as the only systemic therapy available. Lenvatinib is another oral multikinase inhibitor proven to be non-inferior to sorafenib in terms of overall survival for patients with advanced HCC [68]. Lenvatinib has also showed statistically significant improvement in progression-free survival, objective response rate and time to progression [69]. When compared to sorafenib, this new agent presents a similar safety profile [70]. In the latest guidelines, the combination of atezolizumab/bevacizumab or tremelimumab/durvalumab is considered the first line treatment for advanced HCC [71].

Since TACE leads to an upregulation of VEGF and PDGF by causing tumor cell hypoxia, the combination of TACE and sorafenib has been attempted in order to improve clinical outcomes [72]. 

The TACTICS trial compared the efficacy and safety of TACE plus sorafenib (S-TACE) with TACE alone in patients with unresectable HCC. The trial concluded that TACE, when combined with sorafenib, significantly improved progression-free survival over TACE alone (25.2 vs. 13.5 months; *p* = 0.006) [72]. A meta-analysis, including only patients with portal vein tumor thrombus, also came to the conclusion that the combination treatment may improve overall survival, time to progression, and objective response rate when compared to TACE monotherapy [73]. On the contrary, a clinical trial comparing the two treatment strategies in patients with intermediate stage, failed to prove the superiority of the combination treatment over TACE alone [74]. Finally, a different study comparing S-TACE and TACE monotherapy in patients with diffuse recurrence (defined as 10 or more new recurrent nodules) indicated that S-TACE favored those patients in terms of overall survival (24.0 vs. 16.0 months; *p* = 0.044 in patients with late recurrence) [75].

Recently, Kudo, et al. have demonstrated that lenvatinib-TACE sequential therapy markedly improves overall survival when compared with TACE alone in patients with intermediate-stage HCC beyond up-to-seven criteria [76]. A different treatment strategy suggested also by Kudo et al., the ABC Conversion Therapy (atezolizumab/bevacizumab combination therapy followed by surgical resection, RFA, or selective TACE) reports promising results [77]. Since atezolizumab/bevacizumab combination therapy produces marked tumor shrinkage, surgical resection, ablation and TACE become more feasible. Further studies are needed to confirm these findings.

### 4.6. TACE & Immunotherapy

Over the past years, the development of molecular biology and the continuous study of tumorigenesis have led to the introduction of a novel form of therapy in the management of HCC immunotherapy [78]. In particular, various studies have focused on the efficacy of combined therapy with liver-directed therapies, such as TACE and RF. Locoregional methods have shown to modify the local immune environment and release tumor antigens [79]. Therefore, it is presumed that immunotherapy agents can act as adjuvant therapy to prevent recurrence and metastasis [80].

A study evaluating the efficacy of autologous cytokine-induced killer (CIK) cell transfusion in combination with TACE and RFA, concluded that TACE + RFA + CIK group had longer overall survival and progression free survival that TACE + RFA group, with no significant difference at adverse effect frequency between the two groups [81]. A meta-analysis comparing survival rates of HCC patients divided into two groups (TACE + RFA + CIK versus TACE + RFA), came to the conclusion that CIK cell transfusion therapy and locoregional treatments showed a synergistic effect, resulting in longer recurrence-free survival [80]. Finally, a recent analysis studied the addition of immune checkpoint inhibitors (ICIs) at the therapeutic allocation of patients with intermediate or advanced stage HCC treated with TACE and Sorafenib. The conclusion was that TACE + Sorafenib + ICIs patients demonstrated prolonged overall survival and progression free survival rates when compared with the TACE + Sorafenib group [82] (Table 2).

## 5. Conclusions

**Figure 2:**
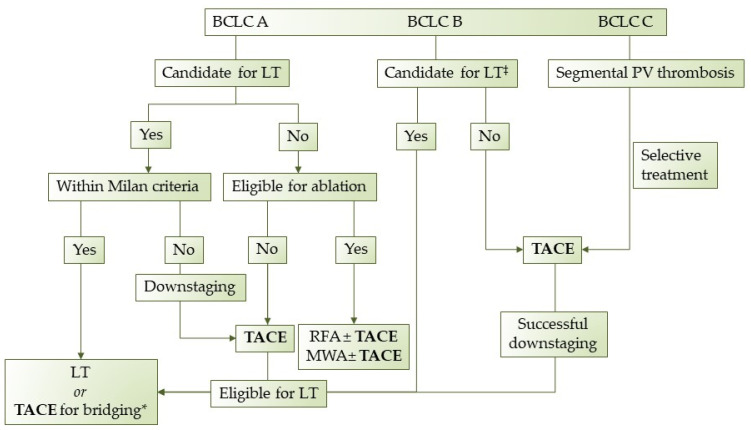
Proposed treatment algorithm regarding TACE in HCC patients. LT: Liver transplantation, TACE: transarterial chemoembolization, RFA: radiofrequency ablation, MWA: microwave ablation, * when in waiting list > 6 months, ^‡^ extended liver transplant criteria (size, AFP).

The purpose of this review was to assess the indications, the optimal treatment schedule, the technical factors associated with TACE, and the overall application of TACE as a personalized treatment for HCC. Even though TACE is currently considered the first-line treatment for patients with HCC in the intermediate stage, recent studies have showed that it can beneficial for patients beyond stage B. Moreover, since BCLC stage B represents a heterogeneous group, not all intermediate-stage HCC patients benefit the same from TACE. Therefore, treatment allocation should be decided by a tumor board of specialists and each HCC patient should receive personalized treatment according to his/her individual features. Unfortunately, in many countries, a tumor board is not available in every hospital and physicians should address virtual boards remotely via telemedicine. Based on our experience and the review of the literature that we conducted, we propose a treatment algorithm regarding TACE procedure in HCC patients (Figure 2). In conclusion, TACE is an established procedure with proven efficacy and known adverse effects and contraindications. Nevertheless, additional studies and clinical trials are warranted to redefine patient selection criteria, introduce new indications, and stratify patients according to their individual prognostic evaluation.

## Figures and Tables

**Table 1:** Prognostic scores. AFP: alpha-fetoprotein, TACE: transarterial chemoembolization, AST: aspartate protein, BCLC: Barcelona Clinic Liver Classification, HCC: Hepatocellular carcinoma.

Score	Parameters Used	Prognostic Value	Demerits
HAP [43]	Albumin, Bilirubin, AFP, Size of dominant tumor	Prognosis of HCC patients undergoing TACE	
ART [44]	Radiological response after the first TACE, increase of serum AST, increase of Child-Pugh score	Differentiation of patients who would benefit from a second TACE	Failed to predict overall survival in patients who received repeated TACE
STATE [45]	Albumin, CRP, Size of the largest tumor, Number of tumors	Identification of patients unsuitable for first-time TACE	
ABCR [46]	AFP, BCLC, Child-Pugh increase, Radiological response	Differentiation of patients who would benefit from a second TACE	Failed to show sufficient prognostic ability to guide the decision-making process regarding subsequent TACE
CHIP [47]	Child-Pugh, number of lesions, HCV-RNA positivity	Stratification of patients within BCLC Stage B	
M-TACE [48]	Bilirubin, INR, CRP, creatinine, AFP, tumor extension	Identification of patients most likely to benefit from TACE	
Six & Twelve [49]	Tumor size, tumor number	Outcome prediction and risk stratification of recommended TACE candidates	Only “ideal” TACE candidates included
Pre-TACE & Post-TACE predict [50]	Tumor size, tumor number, AFP, albumin, bilirubin, vascular invasion, cause, radiological response	Prediction of survival among patients receiving TACE	Calculator needed

**Table 2:** Summary of mentioned studies. cTACE: conventional transarterial chemoembolization, DEB-TACE: drug-eluting beads transarterial chemoembolization, OR: odds ratio, NR: not reported, CI: confidence interval, RR: relative risk, OS: overall survival, PFS: progression-free survival, CR: complete response, RFA: radiofrequency ablation, HR: hazard ratio, MWA: microwave ablation, S-TACE: sorafenib-transarterial chemoembolization, ORR: objective response rate, CIK: cytokine-induced killer, ICI: immune checkpoint inhibitors.

Author	Comparison	Complete Response	Progression-Free Survival	Overall Survival	Safety
Zou et al. [18]	DEB-TACE vs. cTACE	OR 1.38, 95% CI 1.01–1.89	NR	OR 1.41, 95% CI 1.01–1.98	OR 0.59, 95% CI 0.41–0.84 (common adverse effects)
Chen et al. [19]	DEB-TACE vs. cTACE	RR 1.09, 95% CI 0.94–1.25 *p* = 0.25 (not statistically significant)	RR 1.21, 95% CI 1.01–1.44 *p* = 0.005 (1-year PFS)	RR 1.12, 95% CI 1.03–1.23, *p* = 0.007 (1-year OS)	RR 0.87, 95% CI 0.71–1.07 *p* = 0.19 PES (not statistically significant)
Han et al. [20]	DEB-TACE vs. cTACE	OR 3.59, 95% CI 1.48–8.72, *p* = 0.0048	no statistically significant difference	OR 1.92, 95% CI 1.00–3.67, *p* = 0.049 (3-year OS)	no statistically significant difference
Facciorusso et al. [21]	DEB-TACE vs. cTACE	OR 1.21, 95% CI 0.69–2.12 *p* = 0.51 (not statistically significant)	NR	no statistically significant difference	OR 0.85, 95% CI 0.60–1.20 *p* = 0.36 (not statistically significant)
Wang et al. [22]	DEB-TACE vs. cTACE	RR 1.06, 95% CI 0.84–1.34 *p* = 0.17 (not statistically significant)	NR	RR 0.96 95% CI 0.69–1.32 *p* = 0.715 (not statistically significant)	RR 1.22 95% CI 0.87–1.71 *p* = 0.255 (not statistically significant)
Kim et al. [56]	TACE + RFA vs. RFA vs. TACE	TACE-RFA or RFA vs. TACE, *p* < 0.001 (1-year CR)	NR	NR	*p* = 0.006 (in favor of TACE) *p* = 0.009 (in favor of RFA)
Liu et al. [57]	TACE + RFA vs. RFA	NR	*p* < 0.001 (in favor of TACE + RFA)	*p* < 0.001 (in favor of TACE + RFA)	NR
Peng et al. [58]	TACE + RFA vs. RFA	NR	HR 0.575, 95% CI 0.374/0.897 *p* = 0.009	HR 0.525, 95% CI 0.335–0.822 *p* = 0.002	no statistically significant difference
Peng et al. [59]	TACE + RFA vs. RFA	NR	*p* = 0.005 (in favor of TACE + RFA)	*p* = 0.037 (in favor of TACE + RFA)	no statistically significant difference
Zaitoun et al. [60]	TACE + MWA vs. MWA vs. TACE	*p* < 0.0002 (in favor of TACE + MWA)	*p* < 0.001 (in favor of TACE + MWA)	*p* = 0.02 (in favor of TACE + MWA)	no statistically significant difference
Liu et al. [61]	TACE + MWA vs. TACE	RR 2.59, 95% CI 2.09–3.14 *p* < 0.001	NR	RR 2.07, 95% CI 1.67–2.57 *p* < 0.001 (3-year OS)	no statistically significant difference
Yuan et al. [62]	TACE + MWA vs. TACE + RFA	*p* = 0.041 (only for tumor > 5 cm-in favor of TACE + MWA)	no statistically significant difference	no statistically significant difference	no statistically significant difference
Abdelaziz et al. [63]	TACE + MWA vs. TACE + RFA	*p* = 0.01 (only for tumors 3–5 cm-in favor of TACE + MWA)	no statistically significant difference	no statistically significant difference	no statistically significant difference
TACTICS trial, Kudo et al. [72]	S-TACE vs. TACE	no statistically significant difference	*p* = 0.006 (in favor of S-TACE)	NR	NR
Zhang et al. [73]	S-TACE vs. TACE	OR 3.59, 95% CI 1.74–7.39 *p* = 0.0005 (ORR)	NR	HR 0.62, 95% CL 0.51–0.75 *p* < 0.00001	NR
SPACE trial, Lencioni et al. [74]	S-TACE vs. TACE	NR	no statistically significant difference	no statistically significant difference	NR
Yao et al. [75]	S-TACE vs. TACE	NR	*p* = 0.049 (early diffuse recurrence only-in favor of S-TACE)	*p* = 0.011 (in favor of S-TACE)	no statistically significant difference
Kudo et al. [76]	Lenvatinib + TACEvs. TACE	*p* < 0.001 (ORR-in favor of Lenvatinib+ TACE)	HR 0.19, 95% CI 0.10–0.35 *p* < 0.001	HR 0.48 95% CI 0.16–0.79 *p* < 0.01	NR
Huang et al. [81]	TACE + RFA + CIK vs. TACE + RFA	no statistically significant difference	*p* = 0.001 (in favor of TACE+ RFA + CIK)	*p* = 0.001 (in favor of TACE+ RFA + CIK)	no statistically significant difference
Zheng et al. [82]	S-TACE + ICIs vs. S-TACE	*p* = 0.046 (DCR-in favor of S-TACE + ICIs)	*p* < 0.001 (in favor of S-TACE + ICIs)	*p* = 0.012 (in favor of S-TACE + ICIs)	no statistically significant difference

## Data Availability

No new data were created or analyzed in this study. Data sharing is not applicable to this article.
